# Generation and characterization of high affinity human monoclonal antibodies that neutralize staphylococcal enterotoxin B

**DOI:** 10.1186/1476-8518-8-9

**Published:** 2010-12-21

**Authors:** Brian Drozdowski, Yuhong Zhou, Brad Kline, Jared Spidel, Yin Yin Chan, Earl Albone, Howard Turchin, Qimin Chao, Marianne Henry, Jacqueline Balogach, Eric Routhier, Sina Bavari, Nicholas C Nicolaides, Philip M Sass, Luigi Grasso

**Affiliations:** 1 Morphotek Inc., 210 Welsh Pool Road, Exton, PA, USA; 2 U.S. Army Medical Research Institute of Infectious Diseases, Fort Detrick, Frederick, MD, USA

## Abstract

**Background:**

Staphylococcal enterotoxins are considered potential biowarfare agents that can be spread through ingestion or inhalation. Staphylococcal enterotoxin B (SEB) is a widely studied superantigen that can directly stimulate T-cells to release a massive amount of proinflammatory cytokines by bridging the MHC II molecules on an antigen presenting cell (APC) and the Vβ chains of the T-cell receptor (TCR). This potentially can lead to toxic, debilitating and lethal effects. Currently, there are no preventative measures for SEB exposure, only supportive therapies.

**Methods:**

To develop a potential therapeutic candidate to combat SEB exposure, we have generated three human B-cell hybridomas that produce human monoclonal antibodies (HuMAbs) to SEB. These HuMAbs were screened for specificity, affinity and the ability to block SEB activity *in vitro *as well as its lethal effect *in vivo*.

**Results:**

The high-affinity HuMAbs, as determined by BiaCore analysis, were specific to SEB with minimal crossreactivity to related toxins by ELISA. In an immunoblotting experiment, our HuMAbs bound SEB mixed in a cell lysate and did not bind any of the lysate proteins. In an *in vitro *cell-based assay, these HuMAbs could inhibit SEB-induced secretion of the proinflammatory cytokines (INF-γ and TNF-α) by primary human lymphocytes with high potency. In an *in vivo *LPS-potentiated mouse model, our lead antibody, HuMAb-154, was capable of neutralizing up to 100 μg of SEB challenge equivalent to 500 times over the reported LD_50 _(0.2 μg) , protecting mice from death. Extended survival was also observed when HuMAb-154 was administered after SEB challenge.

**Conclusion:**

We have generated high-affinity SEB-specific antibodies capable of neutralizing SEB *in *vitro as well as *in vivo *in a mouse model. Taken together, these results suggest that our antibodies hold the potential as passive immunotherapies for both prophylactic and therapeutic countermeasures of SEB exposure.

## Introduction

*Staphylococcus aureus *is a Gram-positive bacterium responsible for skin, soft-tissue, respiratory, bone, joint, and endovascular disorders, and has potentially lethal effects due to endocarditis, sepsis, and toxic shock syndrome [1]. Virulence for a number of the pathogenic manifestations of *S. aureus *is caused by a handful of toxins produced and secreted by the bacterium, which include among others the toxins responsible for toxic shock syndrome, TSST-1, and *S. aureus *enterotoxins (SEs), which cause food poisoning. About twenty enterotoxins have been described that exhibit and are defined by their emetic activity in primates [2-6]. Enterotoxins are also referred to as superantigens (SAgs) because they bypass antigen processing by forming a bridge between the MHC II molecules on an antigen presenting cell (APC) and the Vβ chain of the T-cell receptor (TCR) causing a massive release of cytokines, such as interferon-gamma (INF-γ) and tumor necrosis factor-alpha (TNF-α). SEB is one of the most studied enterotoxins notoriously associated with food poisoning through ingestion. Symptoms include a rapid onset of fever, intense nausea, vomiting, cramping abdominal pain, and diarrhea. Most cases are self-limited and resolve in 8-24 hours. If aerosolized, SEB could cause severe cases of pulmonary edema and respiratory failure [7,8]. Since it has the potential to be weaponized and used as an incapacitating or lethal agent, the National Institute of Allergy and Infectious Diseases (NIAID) and the Centers for Disease Control and Prevention (CDC) recognize SEB as a category B agent.

Currently, there are no commercial preventative measures or therapies for SEB exposure based on passive (antibodies) or active (vaccines) immunotherapy, despite the fact that multiple attempts to develop therapies have met with various degrees of success. SEB mutants generated by site-directed mutagenesis and lacking superantigenic effects are highly immunogenic in mice and rhesus monkeys, demonstrating their potential as a vaccine for prophylactic intervention [9]. Woody *et al *have studied the vaccine potential of mutant staphylococcal SEB proteins and showed that some were able to elicit a protective antibody response in LPS-potentiated mice [10]. Strategies aimed at disrupting SEs interaction with the immune system include low-molecular antagonist peptides, based on the SEs conserved regions, as well as soluble T-cell receptor that can sequester SEB [11-14]. The use of mouse monoclonal anti-SEB antibodies to study important epitope determinants essential for MHC/TCR binding has led others to explore the use of anti-SEB antibodies for blocking SEB from engaging the immune system [15]. Other notable studies have included a murine toxic shock syndrome toxin 1 (TSST-1)-specific monoclonal antibody (MAb) which crossreacted to SEB by ELISA and partially inhibited SEB-induced T-cell mitogenesis as well as TNFα secretion in human PBMCs in a dose-dependent manner *in vitro *[16]. Also, LeClaire *et al *demonstrated the feasibility of using a passive immunity strategy utilizing SEB-specific MAbs raised in chicken to block SEB-mediated toxicity in Rhesus monkeys [17]. In this study, animals (4/4) that received molar ratios of antibody to toxin of 21:1 and 37:1 survived an aerosolized exposure of approximately 5 LD_50 _of SEB.

Pooled human sera with titers against SEs and TSST-1 were reported to suppress *in vitro *SEB-induced human T-cell proliferation while affinity purified anti-SEB antibodies from the pooled human sera prophylactically protected mice from a SEB lethal challenge [18]. More recently, chimeric mouse-human antibodies with high affinities for SEB were reported to inhibit SEB induced proliferation and cytokine production in both human PBMCs and mouse splenocytes *in vitro *[19]. In this report, we detail the generation and selection of fully human monoclonal antibodies (HuMAbs) specific for SEB derived from human B-cell hybridomas. These antibodies showed biological activity towards SEB *in vitro*. In addition, HuMAb-154, which displayed the highest anti-SEB affinity, exhibited prophylactic as well as therapeutic activity in a mouse model of SEB-induced lethality.

## Materials and methods

### Generation of HuMAbs

Human B-cell hybridomas producing SEB-specific HuMAbs were generated using Human MORPHODOMA^® ^technology as previously described [20]. Briefly, leukopacks were obtained from healthy individuals with anti-SEB serum antibody titers. B cells were isolated from the PBMC population using an EasySep human B cell enrichment kit (StemCell Technologies). B cells were cultured for 7 days in IMDM media (Invitrogen) supplemented with 10% heat-inactivated FBS (JRH BioSciences), 10 ng/ml human IL-2, 10 ng/ml human IL-10 (PeproTech), 2 mM L-glutamine, 0.1 mM nonessential amino acids, 1 mM sodium pyruvate, 55 μM 2-mercaptoethanol (Invitrogen) in the presence of irradiated CHO feeder cells. Cultured B cells were then electrofused with CB-F7 heteromyeloma cells [21] (kind gift from Dr. Roland Grunow, Robert Koch-Institute) using the Cyto Pulse CEEF-50 apparatus (Cyto Pulse Sciences) to generate hybridomas. Individual hybridoma clones were screened by SEB-specific ELISA (Recombinant SEB, highly purified, from Toxin Technology, Inc.) for specific SEB-reactive antibodies. Clones highly reactive with SEB without crossreactivity to the other unrelated antigens were subcloned followed by ELISA screening to confirm retention of SEB specificity. Light and heavy chain genes of lead clones were sequenced and cloned for recombinant expression of corresponding antibodies in CHO cells.

### HuMAb ELISA reactivity to related toxins

Related Staphylococcus enterotoxins (SEs), SEB, SEA, SED, SEC1, TSST-1 (Toxin Technology, Inc.), and tetanus toxoid (Cylex Inc.) were diluted to 0.5 μg/ml in coating buffer (50 mM carbonate-bicarbonate, pH 9.4) and coated in microtiter plates overnight at 4°C. Plates were blocked and ELISA-based screening was performed as for identification of HuMAbs.

### SDS-PAGE and Western blotting

SEB at 500 ng and 20 μg of 293 cell lysates were diluted in SDS sample buffer then subjected to reducing and non-reducing SDS-PAGE on a 4 to 12% gradient gel in MES buffer (NOVEX, San Diego, CA). Electrophoresed proteins were transferred onto nitrocellulose sheets. After transfer, the sheets were blocked with TBS containing 5% milk and 0.1% Tween-20 (5% milk/TBS-T) for 1 hour at room temperature with gentle agitation. Blots were agitated for 1 hour with HuMAbs at 1 μg/ml diluted in 5% milk/TBS-T. The blots were then washed four times (for 5 min each time with agitation) with TBS containing 0.1% Tween-20 (TBS-T), followed by gentle agitation for 1 hour at room temperature with peroxidase-conjugated goat anti-human IgG+M (H+L) Ab (Jackson Immuno Laboratories) (diluted 1:10,000 in 5% milk/TBS-T). The blots were washed again four times and the immunoreactive proteins were visualized using SuperSignal West Femto Luminescent Substrate (Pierce).

### Surface plasmon resonance

SEB was diluted in 10 mM sodium acetate pH 5.5, to a final concentration of 10 μg/ml and coupled to the surface of a research-grade CM5 chip (Biacore, Inc., Piscataway, NJ) by standard amine chemistry (NHS-EDC, Biacore, Inc.), to a level of 50-250 RU of SEB bound. The remaining active sites were quenched using 1 M ethanolamine. A reference flow cell consisting of an activated and quenched surface in the absence of antigen was created and used to normalize readings from injections of anti-SEB-containing samples. Purified HuMAbs were diluted into HBS-EP buffer (Biacore, Inc.) to final concentrations of 50 nM, 25 nM, 12.5 nM, 6.25 nM, 3.13 nM, 1.56 nM and 0.78 nM. The running buffer used was HBS-EP with a flow rate of 30 μl/min. Regeneration of the surface was performed by two injections of 10 mM HCl for 30 seconds at a flow rate of 100 μl/min. The on- (ka) and off-rate (kd) of HuMAbs were determined by observing the signal over time for triplicate injections at each concentration above. Blank injections of HBS-EP were also performed to assess noise, and to normalize injection data. All samples were randomly injected for two minutes using the KINJECT command and the dissociation phase of binding was observed for 10 minutes. The data were fitted to a bivalent analyte binding model, using BiaEvaluation 4.1 software (Biacore, Inc.), as no mass transfer effects were obvious in control experiments. A steady-state binding constant (K_D1_) was derived from the observed on- and off-rates (k_a1 _and k_d1_, respectively) for first-site binding of antibody to SEB.

### *In vitro *toxin neutralization

Human peripheral blood mononuclear cells (PBMCs) were used to determine the ability of HuMAbs to inhibit SEB-induced T-cell cytokine production and measure their *in vitro *EC_50_. PBMCs isolated from blood drawn from healthy volunteers were purified by Ficoll-Paque Plus (Amersham-Pharmacia). Approximately 1 × 10^**5 **^cells in 100 μl of IMDM medium supplemented with 10% heat-inactivated FBS (JRH Biosciences), 2 mM L-glutamine, 0.1 mM nonessential amino acids , 1 mM sodium pyruvate , 55 μM 2-mercaptoethanol (Invitrogen), 5 μg/ml gentamicin (Gibco), were cultured in 96-well flat-bottom tissue culture plates and incubated at 37°C in 5% CO2. Various 4x concentrations of HuMAbs or combination thereof at different ratios were incubated with SEB (4x its *in vitro *ED_50_, data not shown) for 1 h and the mixture subsequently added to the PBMCs (1x final concentration for both HuMAbs and SEB). After 18-22 hours, supernatants were transferred to IFN-γ- and TNF-α-coated ELISA plates (75 μl/well) and assayed using an ELISA kit (R&D System) following the manufacturer's recommended procedure. EC_50 _calculations of HuMAbs were performed using Prism4 (GraphPad Software). The sensitivity limit of the IFN-γ and TNF-α ELISA is 16 pg/ml.

### *In vivo *toxin neutralization

*In vivo *studies were conducted by TransPharm Preclinical Solutions as well as at US Army Medical Research Institute for Infectious Diseases. Female Balb/C mice ordered from Charles River weighing 14-16 g were acclimated to housing conditions and handled in accordance with AUP number TP-06-08. They were 7-8 weeks old on Day 1 of the experiment. The animals were fed irradiated Rodent Diet 5053 (LabDiet™) and water *ad libitum*. Mice were housed in static cages with Bed-O'Cobs™ bedding inside BioBubble^® ^Clean Rooms that provide H.E.P.A filtered air into the bubble environment at 100 complete air changes per hour. All challenges were carried out in the BioBubble environment. The environment was controlled to a temperature range of 71° ± 4°F and a humidity range of 30-70%. All animals were observed for clinical signs at least once daily. All procedures carried out in this experiment were conducted in compliance with all the laws, regulations and guidelines of the National Institutes of Health (NIH) and with the approval of the TPPS Animal Care and Use Committee.

Mice were challenged with various concentrations of SEB (Toxin Technology, FL) to assess the protective activity of HuMAb-154 *in vivo*. The presumptive LD_50 _of SEB in Balb/C mice was 0.2 μg/mouse [18]. SEB challenges of 1, 5, 10, 20, 50, and 100 μg SEB/mouse were premixed with 500 μg of HuMAb-154 then injected intraperitoneally. To spare additional animals from an unnecessary sacrifice, only mice treated with HuMAb-154 were challenged with SEB doses higher than 5 ug of SEB. SEB toxicity was potentiated by the administration of LPS (150 μg/mouse) three hours following the SEB challenge. Challenge controls were injected with SEB alone and LPS alone. Survival was determined after 3 days. Time course studies were conducted to assess the therapeutic activity of a 500 μg dose of HuMAb-154 administered at 0, 0.5 and 1 hr following a challenge of 5 μg and 10 μg of SEB. An LPS potentiating dose of 150 μg/mouse was delivered three hours following the SEB challenge. Survival was determined after 4 days.

### Statistical methods

Data was analyzed in the Prism software package (GraphPad Software, Inc). An unpaired two-tailed t-test was used to analyze *in vitro *neutralization assays while *in vivo *survival curves were analyzed using the Log-rank (Mantel Cox) test.

## Results

### Generation of human monoclonal antibodies targeting SEB

**Figure 1:**
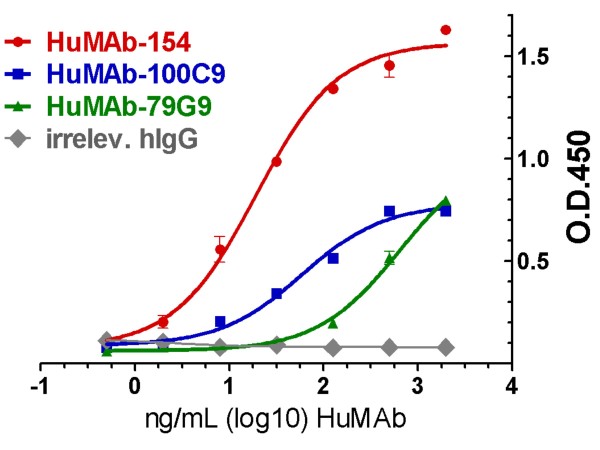
**Binding strength of HuMAbs was tested by ELISA using microplates coated with SEB**. Each point represents the average of triplicate samples ± the SD and the data shown are representative of two or more independent experiments.

Healthy volunteers whose sera showed pre-existing high immune reactivity to SEB were identified by ELISA. B-cells isolated from these selected donors' blood samples were cultured and expanded using an *in vitro *culture system. Expanded B-cells were subsequently fused to myeloma cells to generate a library of hybridomas. We screened the conditioned media produced by these hybridomas by ELISA for reactivity to STEB, an SEB vaccine which is a recombinant and attenuated form of SEB [9]. STEB contains site mutations in the hydrophobic binding loop, polar binding pocket, and disulfide loop (L45R, Y89A, and Y94A, respectively) yet retains its antigenic characteristics [22]. STEB reactive hybridoma clones were further screened against SEB as well as a panel of nonrelated antigens to confirm reactivity and determine specificity. We then subcloned and rescreened SEB-specific hybridomas by ELISA. Three hybridomas, HuMAb-79G9, HuMAb-100C9 and HuMAb-154, met the binding (Figure 1) and selectivity criteria (data not shown) and were chosen for further analyses. We determined the heavy and light chain encoding sequences of these three human IgG1 kappa antibodies and constructed DNA expression vectors that allowed us to recombinantly expressed HuMAbs in mammalian cells for large-scale production.

### Specificity and binding potency of anti-SEB HuMAbs

**Figure 2:**
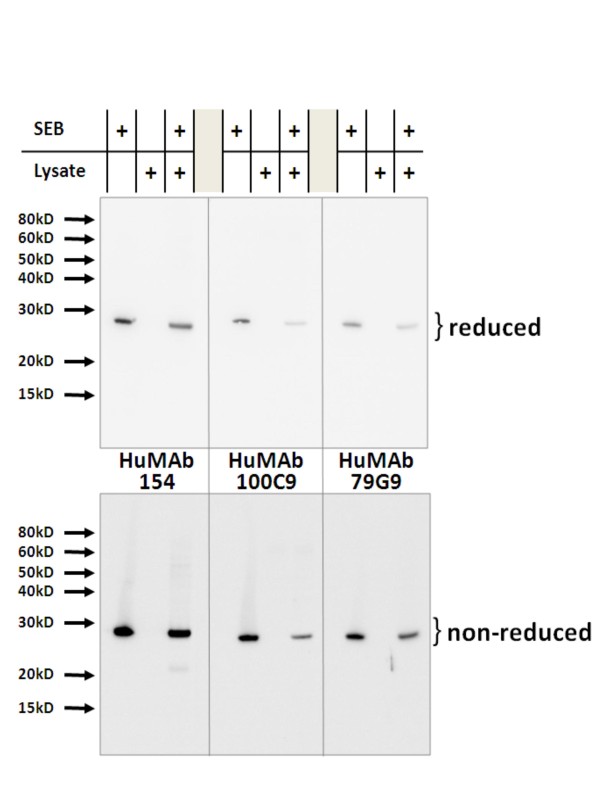
**HuMAb specificity demonstrated by Western blotting**. Human cell lysates (20 μg) spiked with SEB (0.5 μg) were blotted under reducing and non-reducing conditions then probed with HuMAbs. HuMAbs bind only SEB within cell lysates without binding any of the lysate proteins.

Western blotting was used to address whether the HuMAbs reacted non-specifically to cellular proteins. Human cell lysates were spiked with SEB, loaded on SDS-page under reducing and non-reducing conditions, and then probed with each HuMAb. All three HuMAbs recognized the 28 kD SEB in the cell lysate without binding any other cellular protein (Figure 2) demonstrating the specificity of these HuMAbs.

In addition, we carried out surface plasmon resonance (SPR) analyses to determine the binding kinetics of each HuMAb. SEB was immobilized on a CM5 chip followed by injections at various concentrations of HuMAb. Using a bivalent binding model, we determined steady-state binding (K_D1_) constants. HuMAb-154 displayed the strongest affinity (K_D1 _of 290 pM) (Table 1). The different binding strengths measured by SPR correlated well with the binding values as determined by antigen-specific ELISA (Figure 1). Additional SPR analysis using a competitive binding format revealed that HuMAb-154 and HuMAb-100C9 shared the epitope or at least have epitopes in close proximity to one another since HuMAb-100C9 cannot bind SEB that has been saturated with HuMAb-154 (data not shown).

**Table 1:** Binding kinetics of HuMAbs as determined by surface plasmon resonance

HuMAb	k_a1_	k_d1_	K_D1_(nM)
**79G9**	9.56E+03	2.39E-04	25.0
**100C9**	207E+03	11.3E-04	5.46
**154**	93.2E+03	0.27E-04	0.29

### HuMAbs neutralize SEB-induced cytokine production by human lymphocytes

**Figure 3:**
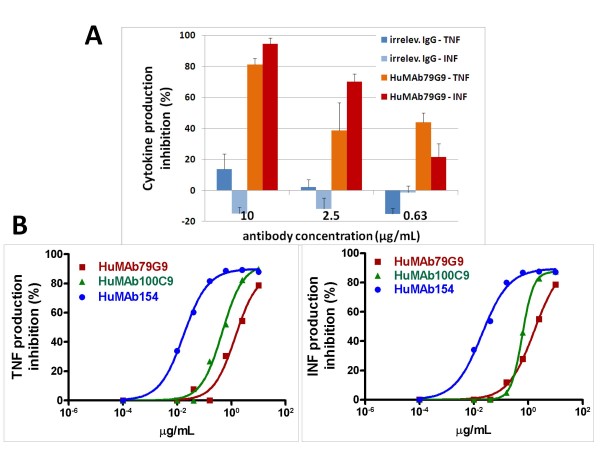
**Assessment of HuMAbs *in vitro *neutralization activity against SEB**. A) Dose-dependent cytokine production inhibitory activity of HuMAb-79G9 as compared to an irrelevant IgG1κ. Each bar represents the average of triplicate samples ± the SD and the data shown are representative of two or more independent experiments. At all antibody concentrations for each cytokine, % inhibition by HuMAb-79G9 was significant (*P *< 0.05) as compared to % inhibition by an irrelevant hIgG. B) Comparison of dose-dependent cytokine production inhibitory activity of three lead HuMAbs. Each point on each line represents the average of triplicate samples ± the SD and the data shown are representative of two or more independent experiments.

To examine the biological activity of HuMAbs *in vitro*, a cell-based assay was employed that measures inhibitory effects on SEB-induced secretion of proinflammatory cytokines by human peripheral blood lymphocytes. Primary human T-cells stimulated with SEB *in vitro *will upregulate the secretion of cytokines including INF-γ and TNF-α, which *in vivo *mediate SEB toxicity. Secretion levels of these cytokines can be monitored using INF-γ- and TNF-α-specific ELISAs. An irrelevant human IgG1κ had no significant inhibitory activity on SEB-induced cytokine production whereas HuMAb-79G9 showed a dose-dependent inhibitory activity (Figure 3A). In subsequent experiments, it was observed that each HuMAb could block SEB-induced activation of human T-cells whereby HuMAb-154 showed the highest potency (Figure 3B), suggesting a correlation between high affinity and potency. It was also observed that HuMAbs alone were not cytotoxic to PBMCs or induced INF-γ and TNF-α secretion as compared to the medium control (data not shown). To define accurately the 50% effective dose (EC_50_) of HuMAb-154, several experiments were conducted using independent batches of purified HuMAb-154. Regardless of the batch used, operator, or the cytokine analyzed, the EC_50 _of HuMAb-154 always remained below 1 ng/mL (Table 2).

**Table 2:** HuMAb-154 lot comparison of inhibitory activity measured by EC_50_

Operator	HuMAb-154	^a^TNFα EC_50_	INFγ EC_50_
1	Lot #1	0.54	0.44
2	Lot #2	0.52	0.49
1	Lot #1	0.56	0.48
2	Lot #2	0.52	0.50
1	Lot #1	0.52	0.51
2	Lot #2	0.64	0.50

### Crossreactivity of HuMAb-154 to other bacterial toxins

**Figure 4:**
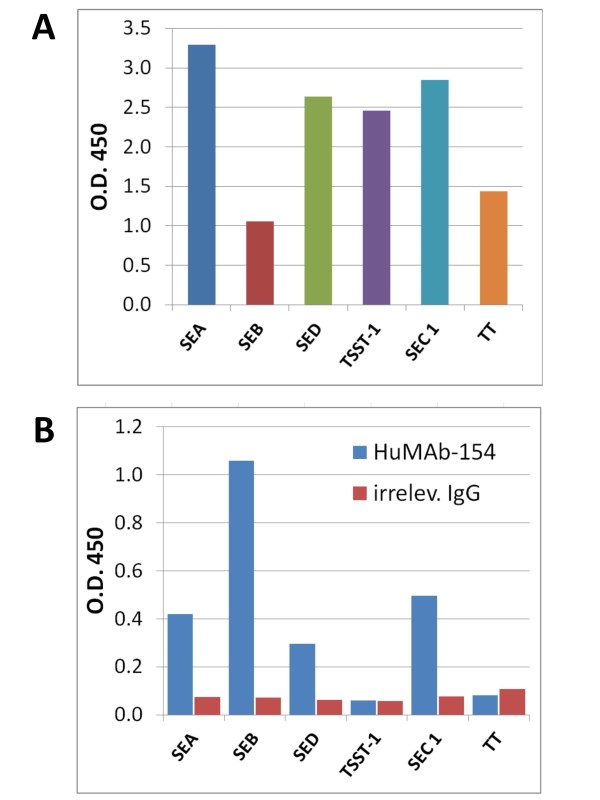
**Cross-reactivity of HuMAb-154 to other bacterial toxins**. A) Toxin-specific control Abs. B) HuMAb-154 and irrelevant hIgG reactivity against the panel of related toxins.

To test whether HuMAb-154 crossreacted to other bacterial toxins, a panel comprising different staphylococcal toxins (SEA, SEB, SED, TSST-1, and SEC1) and the less related tetanus toxin (TT) were screened by ELISA. Toxins were readily detected by the corresponding toxin-specific control antibodies (Figure 4A), thus confirming that all toxins had been efficiently coated on the ELISA microplate. Under these conditions, HuMAb-154 showed the highest reactivity to SEB while having some cross-reactivity to other enterotoxins (SEA, SED, and SEC1, Figure 4B) but not to TSST-1 or TT toxins. An irrelevant human IgG1 did not react with any of the toxins (Figure 4B) confirming the specificity of our assay. HuMAb-154 reactivity to SEC1 is plausible being that SEB and SEC1 share a high degree of amino acid homology whereas SEA and SED show a lower degree of amino acid homology [23,24]. The reactivity of HuMab-154 to SEA and SED is unclear although crystal structure analyses have shown that SEs have similar protein folds despite very different amino acid sequences [25-27].

### Prophylactic administration of HuMAb-154 confers survival of mice exposed to lethal doses of SEB

**Figure 5:**
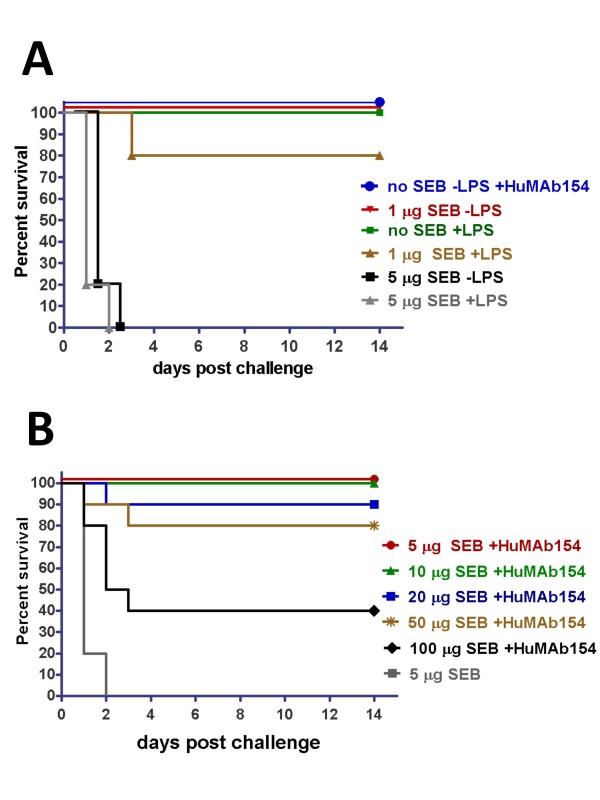
**Prophylactic administration of HuMAb-154 protected mice exposed to lethal doses of SEB challenge**. A) Kaplan-Meier survival chart demonstrates dose escalation of SEB in LPS prime model to find the minimal dose of SEB to reach 100% mortality (*n *= 5). B) Effect of HuMAb-154 at a fixed dose (500 μg) in the protection of LPS primed mice from an escalating SEB challenge. Each HuMAb-154 group contained an *n *of 10 while the untreated contained an *n *of 5. Survival of each SEB challenge group that was administered HuMAb-154 was significant (*P *< 0.015) as compared to the 5 μg SEB challenge group that was not administered HuMAb-154.

Because HuMAb-154 exhibited the best affinity and potency *in vitro*, we focused on this HuMAb for *in vivo *efficacy testing. An LPS-potentiated mouse model [18,28] reported an LD_50 _of ~0.2 μg of SEB. Using the same model, we observed that control groups with LPS alone and HuMAb-154 alone had no lethal or toxic effects on mice (Figure 5A). An LPS potentiating effect at five fold of the reported LD_50 _(1 μg of SEB) was observed which resulted in 20% mortality as compared to no mortality with 1 μg of SEB alone (Figure 5A). A challenge of 5 μg of SEB resulted in 100% mortality regardless of whether LPS was administered or not. At this SEB dose level, most animals died within 24 hours post challenge and the remainder died within 48 hours. In contrast, 500 μg of HuMAb-154 premixed with SEB conferred survival of mice at all SEB dose levels tested (14 days of observation period). HuMAb-154 prophylactic effect was dose-dependent: 100% survival at up to 10 μg of SEB, while the percentage of surviving animals gradually decreased as the doses of SEB increased beyond 10 μg of SEB (Figure 5B). Nonetheless, even at the highest challenge dose of 100 μg, 40% survival of the animals treated with HuMAb-154 was observed.

### HuMAb-154 treatment following SEB challenge improves survival of mice

**Figure 6:**
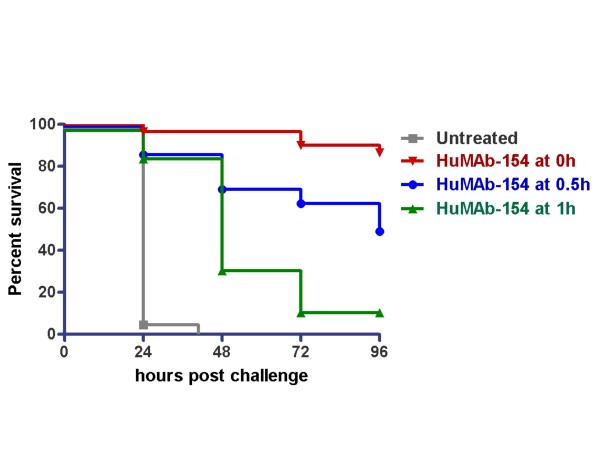
**Kaplan-Meier survival chart showing HuMAb-154 treatment following SEB challenge improves survival of mice**. Time to death was delayed in mice treated with HuMAb-154 shortly after SEB challenge. Mice were challenged with 10 μg of SEB and HuMAb-154 was administered at time points following SEB challenge as indicated. Data are cumulative results of three studies (*n *= 30 for each group). Survival of each HuMAb-154 treatment group was significant (P < 0.0001) as compared to the untreated group.

Using the same Balb/C mouse model described above, additional studies were conducted to determine the therapeutic effects of HuMAb-154 administration following SEB challenge. In this model, 500 μg of HuMAb-154 was injected at different time points after SEB injection. In untreated animals, there was 100% mortality at 10 μg of SEB, whereby 14 of 15 animals in three independent experiments died within 24 hours after SEB challenge and the remaining mouse died within 48 hours (Figure 6). When HuMAb-154 was administered immediately after SEB challenge (0 hours), an average of 86% of the animals survived the challenge (26 of 30 mice in three independent experiments). When HuMAb-154 was administered 30 minutes or 1 hour after SEB challenge, 50% (15 of 30) or 13% (4 of 30) of mice, respectively, survived the challenge while delaying death in the remaining animals. These results suggest that HuMAb-154 can potentially be administered after a lethal or incapacitating dose of SEB and still be able to prevent or delay death.

## Discussion

Antibody technology has been proven as an effective means of blocking SEB-induced pathologies [17,18,29,30]. Furthermore, antibody therapeutics are a well understood segment of the current pharmaceutical industry with many monoclonal candidates already on the market or in the pipeline for several disease indications, and represent a better option over small peptide-based approaches for neutralizing SEB due to their longer half-lives in sera, higher affinities, and reduced immunogenicity. Sources of antibodies usually include (a) animal plasma or serum (antisera); (b) human serum from immunized or convalescent individuals; (c) mammalian cells producing rodent monoclonal antibodies (hybridomas), rodent-human chimeras or humanized antibodies; or (d) mammalian cells producing fully human MAbs.

Animal antisera have been used since the early part of the 20th century to treat toxins like tetanus and diphtheria. The advantages of this approach include its low cost and effectiveness. Disadvantages include potential toxicity upon first and especially on second injection due to the foreign nature of the animal product, which may cause an immune response in humans (serum sickness). Hyperimmune human sera are widely used (e.g. varicella, hepatitis B, CMV and rabies) and are safe and effective. The challenges with such a strategy applied to the SEB case include i) the identification of a large number of human subjects with SEB-specific antibodies, ii) pathogen testing, pooling, storing and processing of the sera to make a safe drug product, and iii) the batch-to-batch variation in the product.

Rodent and rodent-human chimera MAbs can be produced in large scale, but since they retain a significant rodent (foreign) portion, they can have the same immunogenicity as animal antisera. Humanized MAbs retain 5-10% of rodent material. Thus, the immunogenicity of these molecules is reduced but some risks may remain. Because human MAbs do not contain any foreign sequences, they represent the first choice for safe drugs that can be reproducibly manufactured in large scale and indefinitely.

To this goal, we have successfully generated human MAbs that are capable of neutralizing high doses of SEB both *in vitro *and *in vivo*. Our lead antibody HuMAb-154 was selected for further characterization because of its high affinity and *in vitro *high potency and was tested in mouse models of SEB-induced lethality. HuMAb-154 could protect mice prophylactically from a challenge up to 100 μg of SEB injected intraperitoneally, as well as therapeutically, conferring significant protection when administered 30 minutes after SEB challenge. Even though it lost most of its protective effect when administered one hour after SEB challenge, HuMAb-154 significantly delayed the time to death in some animals. It is known that the onset of the SEB-induced toxicity is very rapid and death occurs within 24 hours in most LPS-potentiated mice as reported in other studies [9]. In an LPS-independent mouse model of airway exposure (a route thought to be relevant for mass delivery of SEB in the human population), transgenic mice bearing the human leukocyte antigen-DQ8 died as late as 6 days after 15 μg of aerosol SEB challenge [31]. This evidence further emphasizes the aggressive nature of the SEB toxicity observed in our Balb/C mouse model and indicates that the window of opportunity for therapeutic intervention in this model is very short.

The features of the toxicity induced by aerosolized SEB are not well understood due to the rarity of natural exposures in human. However, it is conceivable that non-human primate models better mimic the progression of the toxicity predicted in humans and may offer a wider window for therapeutic intervention. We will therefore explore these models to further test HuMAb-154 efficacy *in vivo *for both prophylactic and therapeutic countermeasures of SEB exposure.

## Conclusions

There are still no readily available therapeutics to counteract the effects of SEB intoxication, whether the exposure is a result of food poisoning or exposure to a weaponized (aerosol) form of SEB. To this end, we have generated fully human, high-affinity SEB-specific antibodies with potent biological activity towards SEB. This report details the characterization of these antibodies thus providing detailed information for continued study to move these antibodies forward as potential therapeutics.

## Competing interests

The authors declare that they have no competing interests.

## Authors' contributions

BD and YZ carried out the study planning and execution, coordination of mouse studies, statistical analysis and manuscript preparation. BK, JS and YYC sequenced Ig genes and cloned for recombinant expression. EA measured HuMAb affinities. HT purified HuMAbs. QC oversaw the HTS of hybridomas. MH cultured hybridoma cell lines. JB was responsible for HTS of hybridomas. ER oversaw the purification of HuMAbs. SB contributed scientific advisory. NCN and PMS oversaw the general planning, design and implementation of this project. LG participated in the study planning, coordination of mouse studies, manuscript preparation. All authors read and approved the final version of the manuscript.
